# Influence of different rehabilitative aerobic exercise programs on (anti-) inflammatory immune signalling, cognitive and functional capacity in persons with MS – study protocol of a randomized controlled trial

**DOI:** 10.1186/s12883-019-1267-9

**Published:** 2019-03-08

**Authors:** Niklas Joisten, Annette Rademacher, Wilhelm Bloch, Alexander Schenk, Max Oberste, Ulrik Dalgas, Dawn Langdon, Daniel Caminada, Mette-Triin Purde, Roman Gonzenbach, Jan Kool, Philipp Zimmer, Jens Bansi

**Affiliations:** 1 Department of Molecular and Cellular Sport Medicine, Institute of Cardiovascular Research and Sports Medicine, German Sport University Cologne, Am Sportpark Müngersdorf 6, 50933 Cologne, Germany; 2 Deparment of Neurology, Kliniken-Valens, Rehabilitationsklinik-Valens, Taminaplatz 1, 7317 Valens, Switzerland; 3 Department of Public Health, Section of Sport Science, Århus University, Dalgas Avenue 4, 8000 Århus C, Denmark; 4 Royal Holloway University of London, Egham, TW20 0EX Surrey UK; 5 labormedizinisches zentrum Dr Risch, Lagerstrasse 30, 9470 Buchs, Switzerland; 6 Division of Physical Activity, Prevention and Cancer, German Cancer Research Center (DKFZ), Im Neuenheimer Feld 581, 69120 Heidelberg, Germany

**Keywords:** Multiple sclerosis, Rehabilitation, Exercise, High-intensity interval exercise, Immune signalling, Inflammation, Kynurenine pathway, Cognition

## Abstract

**Background:**

Studies have shown positive effects of therapeutic exercise on motor- and cognitive function as well as on psychosocial outcomes in persons with multiple sclerosis (MS). A reduction of inflammatory stress through physical exercise has been suspected as one key mechanism, mediating the positive effects of exercise in the context of MS. The primary objective of this trial is to investigate the acute and chronic effects of different exercise modalities on (anti-)inflammatory immune signalling as well as on cognitive and functional capacity in persons with MS.

**Methods:**

A two armed single-blind randomized controlled design will investigate 72 persons with relapsing remitting or secondary progressive MS (EDSS 3.0–6.0), during 3 weeks of inpatient rehabilitation. Participants will be randomized into either a high-intensity interval training (HIIT) or a moderate continuous training group; the latter represents the local standard therapy (ST). Both groups will exercise 3x per week. The HIIT group will perform 5 × 1.5-min high-intensive exercise bouts at 95–100% of their maximum heart rate (HR_max_) followed by active breaks of unloaded pedalling (60% HR_max_) for 2 min. In contrast, the ST group will exercise for 24 min continuously at 65% of HR_max_. The proportion of circulating regulatory T-cells will be measured as primary outcome. Secondary outcomes comprise numbers and proportions of further immune cells including Th17-cells, soluble factors ((anti-) inflammatory cytokines, tryptophan metabolites), endurance capacity, cognitive performance, processing skills for activities of daily living, fatigue, depression and healthcare-related quality of life. Outcomes will be assessed before (T_0_) and after (T_3_) the 3-week exercise intervention program. Blood samples of T_0_ will be taken immediately before the first exercise session. Additionally, blood samples for the soluble factors will be collected immediately after (T_1_) and three hours (T_2_) after the first exercise session of each group.

**Discussion:**

This study will be the first to investigate both acute and chronic effects of aerobic exercise on immune function and disease associated biomarkers in persons with MS. Combining biological analyses with cognitive and functional capacity assessments may contribute to a better understanding of responses to rehabilitative training, needed to improve exercise recommendations for persons with MS.

**Trial registration:**

This trial was prospectively registered at ClinicalTrials.gov (NCT03652519; 29 August 2018).

## Background

Several studies have shown positive effects of therapeutic exercise interventions on motor- and cognitive function as well as on psychosocial outcomes in persons with MS [1, 2]. Moreover, results from animal models and preliminary data in humans suggest that exercise could serve as a disease modifying supportive treatment [3–5]. Although the knowledge about the underlying biological mechanism provides hypotheses for improving training interventions and exercise recommendations for persons with MS, little controlled research has been conducted. As MS was shown to be accompanied or mediated by neuro-inflammation [6, 7], previously reported anti-inflammatory effects of physical exercise and training [8] have been hypothesized as a possible biological link contributing to the positive effects of exercise in persons with MS [9–11]. Furthermore, a chronic systemic inflammation is widely accepted as a common risk factor for many other chronic diseases (diabetes, cancer, Alzheimer’s disease, Parkinson’s disease, depressions) [12–14] and has further been observed in several patient populations suffering from fatigue and cognitive impairments [15]. It has been suggested that anti-inflammatory properties of exercise depend on two key mechanisms. First, regular exercise reduces visceral fat mass, a major source of inflammation, also known as “metflammation” (indirect effect) [16, 17]. Second, regular exercise and higher physical fitness levels are associated with increased numbers and proportions of circulating anti-inflammatory regulatory T-cells (T_regs_) (direct effect) [18]. T_regs_ suppress an over-activation of the immune system and produce anti-inflammatory cytokines (e.g. Interleukin (IL)-10 and Transforming Growth Factor beta (TGF-β)). The chronic increase in T_regs_ is interpreted as the body’s response to repetitive short-term inflammatory stimuli as they appear during and shortly after each bout of physical exercise, depending on exercise type, intensity and duration.

Interestingly, studies have shown that inflammatory stimuli, as they appear after acute exercise, activate the breakdown of the amino acid Tryptophan (TRP) within the Kynurenine (KYN) pathway [19]. In detail, inflammatory signals are known to induce the initial enzyme of this metabolic path (indoleamine 2,3-dioxygenase-1 (IDO1)) in various tissues, leading to a breakdown of TRP to KYN [20]. Moreover, acute exercise elevates circulating cortisol levels [21], a well described activator of the liver specific tryptophan 2,3-dioxygenase (TDO) [22], representing an isoenzyme of IDO1. KYN itself has previously been described to stimulate the differentiation of T_regs_ and to inhibit the activation of pro-inflammatory immune cells [23, 24]. Therefore, an acute exercise-induced activation of the KYN pathway may represent a possible explanation for chronic elevated numbers of T_regs_ together with further anti-inflammatory effects of exercise.

Besides the immune-modifying properties, chronic activation of the KYN pathway seems to play a pivotal and common role in the pathogenesis of several neurodegenerative disorders including MS [25, 26]. Elevated levels of KYN within the central nervous system lead to an accumulation of its neurotoxic metabolite quinolinic acid (QUIN) [27]. Recently, it was shown that physical exercise leads to a peripheral breakdown of KYN to kynurenic acid (KYNA) by inducing kynurenine aminotransferases (KATs) in muscle tissue [28]. In contrast to KYN, KYNA is unable to cross the blood brain barrier (BBB). Thus, physical exercise reduces the neurotoxic component of the KYN pathway, that was further described to be involved in cognitive dysfunction in neurological diseases [29, 30].

The described chronic anti-inflammatory effects of exercise may have further implications for MS. Recently we have shown that in contrast to moderate continuous training, intensive interval training chronically reduces serum levels of matrix metalloproteinase (MMP)-2 [9, 11]. MMP levels are upregulated by inflammatory stimuli and are further suspected to be involved in the pathogenesis of MS. More precisely MMPs increase the permeability of the BBB, leading to an alleviated infiltration of immune cells into the central nervous system [31].

In order to give more detailed recommendations for rehabilitative training programs it will be of major relevance to compare the influence of varying exercise modalities. Against this backdrop, the overall objective of the present study is to investigate acute and chronic effects of two different aerobic exercise programs on the immune function in persons with MS during rehabilitation. Acute effects are defined as alterations between immediately before, directly after and 3 h after the first exercise session. Chronic effects represent potential changes of resting levels over a 3-week training period. Additionally, the use of patient-oriented outcomes (e.g. cognitive performance, fatigue, depression) will provide new insights into potential associations with biological markers.

## Methods/design

**Fig. 1:**
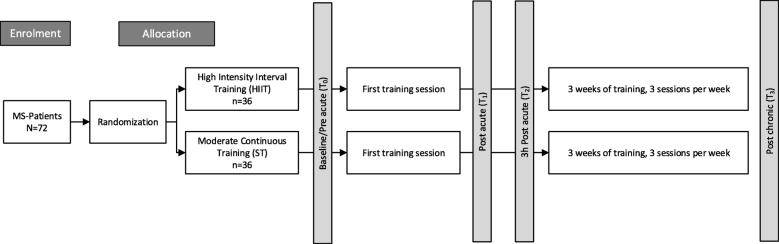
Experimental design of the planned study. Baseline assessments will be conducted before the first training session (T_0_) and will be used as baseline value for both acute and chronic exercise training effects. Measurement time points T_1_ (immediately after first training session) and T_2_ (3 h after first training session) are implemented to examine the acute effects of each exercise modality on soluble factors. All assessments will be repeated 48 h after the last exercise session (T_3_) to investigate effects of chronic training following the 3-week exercise intervention

A two-armed single-blind randomized controlled trial design will be adopted. Prior to inclusion, participants will be asked to provide written informed consent. Participants will be randomized into two different training groups performing either high-intensive interval (HIIT), or moderate continuous (ST) aerobic exercise training on a bicycle ergometer over a period of 3 weeks. Both groups will be assessed on the cardiopulmonary exercise test (CPET) before and after the intervention period. The training performed within both groups will be supervised by independent physiotherapists who will be blind to all other study assessments. The principal investigator will complete CPET and data analysis blinded to the training condition. Participants will not be blinded regarding their individual training intensity. The study design will contain four measurement time-points, considering both acute and chronic effects of each exercise training modality (see Fig. 1). All outcome parameters will be assessed at baseline within a period of 48 h before the first exercise session (T_0_) and at the end of the 3-week exercise training intervention, 48 h following the last training session (T_3_). Blood samples at baseline (T_0_) will be taken immediately before the first exercise session. Outcome changes between T_0_ and T_3_ will indicate the effects of chronic exercise training. Additionally, blood samples will be taken immediately after completion of the first exercise session (T_1_) and 3 h after completion of the first exercise session (T_2_) to provide further information on the acute effects of different exercise modalities on (anti-) inflammatory factors, TRP metabolites and BBB markers. The measurement time point T_2_ is implemented in the study design to examine the kinetics of soluble factors in response to a single-bout of exercise.

This study was approved by the regional ethics committee (EKOS) (EKOS18/96; Project ID: 2018–01378).

### Participants and randomization

Participants will be consecutively recruited on the day of admission for inpatient rehabilitation in the Valens Rehabilitation Center (Switzerland). They will be screened for study inclusion during a 12-month period. Key inclusion criterion for the participants’ eligibility will be a definite MS diagnosis (according to the revised McDonald criteria [32]) with relapsing remitting or secondary progressive MS phenotype and an Expanded Disability Status Scale (EDSS) [33] score between 3.0 and 6.0 (inclusive). Participants will be excluded if they experience acute severe relapses during the 3-week study, are given immune-modulating medication the day of CPET at baseline or T_3_, or withdraw their consent to participate. Detailed information for in- and exclusion criteria are shown in Table 1.

**Table 1:** In- and exclusion criteria

INCLUSION	EXCLUSION
• *Relapsing remitting* or *secondary progressive MS*	• Pregnant or breast feeding women
• *EDSS: 3.0 – 6.0*	• Women planning to become pregnant during study period
• *Age > 21 years*	• Concomitant diseases (internistic, neurological or orthopaedic) limiting the execution of the intervention and assessments or affecting study endpoints
• Informed *consent* as documented by signature	• Drug or alcohol abuse
	• Problems of understanding (not German-speaking), psychological disorders, dementia etc. affecting the ability to understand study course and execute study instructions
	• Persons who already participated in the current study
	• Enrolment of persons employed for study execution, family members of the investigator and himself
	• Acute melanoma and cancer diseases under treatment of chemotherapy or radiation therapy

Participants will be randomly allocated (1:1) to one of the two training programs. Randomization will be conducted using “Randomization-In-Treatment-Arms” software (RITA, Evident, Germany). Randomization will follow the minimization procedure according to Pocock and Simon (for review see Scott et al. [34]) using disease severity (EDSS score), fatigue, age and level of cardiorespiratory fitness as factors for stratification.

### Study intervention

Treatment in both groups consists of specific endurance exercise modalities. Both groups will exercise 3 times per week over a period of 3 weeks on a bicycle ergometer. Exercise sessions will be supervised by physiotherapists. Exercise intensity will be regulated based on HR_max_ determined during the initial CPET. Exercise sessions in both groups will include a warm-up and a cool-down period at low intensity (50% HR_max_) for 3 min each.

#### Experimental intervention group (HIIT)

The experimental intervention group will receive a HIIT consisting of physiologically defined heart rate controlled cycling with 80–100 rpm (rpm) at 95–100% of HR_max_. The HIIT group will perform 5 × 1.5-min high-intensive exercise bouts at 95–100% of their HR_max_ followed by active breaks of unloaded pedalling over 2 min with the aim to achieve 60% of HR_max_.

#### Control group (ST)

In contrast, the ST group will exercise for 24 min continuously at 65% of participants’ HR_max_. (60–70 rpm). The ST group exercise modalities follow the standard therapy regime of the Valens clinic.

### Outcomes and assessments

#### Primary outcome

The primary outcome of this study is the change in proportions of circulating T_regs_ over the 3-week training period (T_0_ to T_3_). Blood samples will be taken by doctors or specialised nurses, via vein puncture from the antecubital vein in a seated position at T_0_ immediately before the first training session and at T_3_ after 3 weeks of training. Patients will be given 10 min between sampling and start of the exercise session to recover from any inconvenience caused by the vein puncture. All blood samples will be taken in K2 EDTA-tubes and Buffy-coat will be collected by centrifugation. Buffy-coat will be stored at − 150 °C until study completion. All samples will be analysed using flow cytometry to evaluate the proportions of T_regs_. T_regs_ will be assessed as CD3^+^CD4^+^CD25^+^ and CD127^dim^.

#### Secondary outcomes

All secondary outcomes will be assessed at baseline (T_0_) and after the 3-week training intervention (T_3_). To examine acute effects of the first exercise session blood samples will be additionally taken straight after completion of the first exercise session during cool down (T_1_) with the participant still seated on the ergometer as well as three hours after completion of the first exercise session (T_2_).

**Fig. 2:**
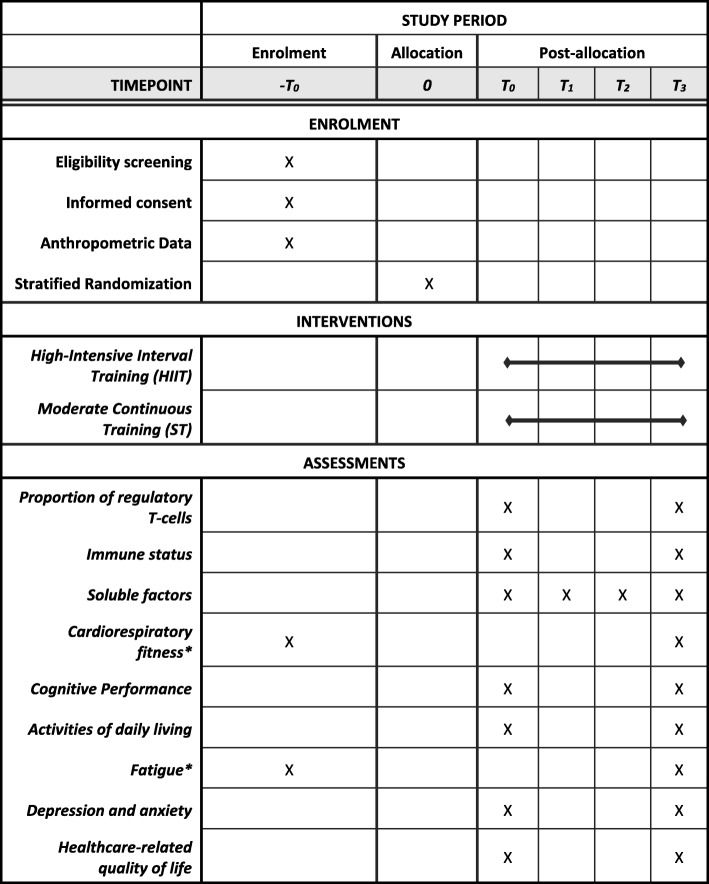
Spirit diagram depicting the schedule of enrolment, interventions and assessments. T_0_ = baseline assessment before the first exercise session; T_1_ = immediately after the first exercise session; T_2_ = three hours after the first exercise session; T_3_ = 48 h after the last exercise session. * Cardiorespiratory fitness and fatigue score are used as stratification factor and will be assessed before allocation

Blood samples for secondary outcomes at T_0_ and T_3_ will be taken at the same time as those of the primary outcome by using additional K2 EDTA- tubes for buffy-coat and plasma isolation. Plasma will be stored at − 80 °C until study completion. Moreover, cognitive and functional capacity data will be collected to identify potential associations with biomarker changes and to get better insight into the impact of different exercise training modalities. For a detailed overview of all outcomes and its assessments during the study period please see Fig. 2. The following will be captured as secondary outcomes:

##### Immune status

An immune status including numbers and proportions of lymphocytes, T-cells (CD3^+^), NK-cells (CD3^−^CD56^+^) and B-cells (CD3^−^CD19^+^) will be assessed at T_0_ and T_3_. Furthermore, the T-cells subpopulations cytotoxic T-cells (CD3^+^CD8^+^), T-helper cells (CD3^+^CD4^+^), TH-17 cells (CD3^+^CD4^+^CD161^+^CCR6^+^), naïve T-cells (CD4^+^CD45RA^+^) as well as memory T-cells (CD4^+^CD45RO^+^) will be identified. Additionally, numbers of T_regs_ (CD3^+^CD4^+^CD25^+^ and CD127^dim^) will be assessed. Numbers and proportions will be determined by using flow cytometry. Gating strategies will be applied as reported by Zimmer et al. and Wenning et al. [35, 36].

##### Soluble factors

Inflammatory (Tumor necrosis factor-alpha, Interferon-gamma, IL-6, IL-17) and anti-inflammatory (IL-10, TGF-β) soluble factors as well as Tryptophan metabolites (TRP, KYN, KYNA, QUIN) that are known to be produced or secreted in response to exercise and that are further suspected to modify immune homeostasis and BBB function (MMP-2, MMP-9) through their inflammatory and anti-inflammatory properties will be measured at all time-points (T_0_ – T_3_). Therefore, plasma levels of TRP, KYN, TNF-alpha, IFN-gamma, IL-6, MMP-2, MMP-9, IL-10, TGF-beta and IL-17 will be determined using Enzyme-linked immuno-sorbent assay (ELISA) according to manufactures instructions. Levels of KYNA and QUIN will be assessed by high-performance liquid chromatography.

##### Cognitive performance

Cognitive performance will be assessed at T_0_ and T_3_ with the Brief International Cognitive Assessment for Multiple Sclerosis (BICAMS) [37] and a Go/No-Go test paradigm of the test battery of attention performance (*TAP*) [38]. The BICAMS is a battery involving three brief tests to assess the main cognitive domains vulnerable to MS: information processing speed, verbal and visual memory. The tests are the Symbol Digit Modalities Test (SDMT), the Verbal Learning and Memory Test (VLMT) and the Brief Visuospatial Memory Test revised (BVMT-R). Parallel versions of each test will be applied at T_3_.

The SDMT assesses information processing speed. Digits from one to nine are each paired with one of nine abstract symbols. The test consists of 110 of these nine symbols arranged in rows pseudo-randomly. The participant is instructed to name the corresponding number of each symbol, over 90 s after a training part of ten symbols is completed. The overall execution of the SDMT which includes the instructions, the training and testing parts takes 5 min.

The VLMT consists of a 15-item word list which is read aloud to the participant who is then asked to recall as many words as possible. The VLMT compromises 5 trials of which each includes a reading of the list and a repetition of the words by the participant. A defined order for repetition does not exist. The VLMT takes 5–10 min.

The BVMT-R compromises six abstract geometric figures located on a 2 × 3 array. The stimulus sheet is shown to the participant for 10s. After its removal the participant is asked to draw as many figures as possible from memory in the correct location, shape and proportion. There are three learning trials of 10s each after which the participant is required to draw the symbols. The drawing of the trial before is not visible for the participant during the other trials.

In addition to the BICAMS, the Test of attentional Performance (TAP), a classical Go/No-Go test paradigm will be completed [39]. Go/No Go paradigm tests are used to assess the ability to perform an appropriate reaction under time pressure and to simultaneously inhibit a formed behavioural response (automatic inhibition) [40]. In this paradigm, the focus of attention is directed to predictably occurring stimuli that require a selective reaction (to react if “x” appears and not to react if “+” appears, during 2 min). The TAP takes approximately 5 min.

##### Activities of daily living (ADL)

The performance of individuals’ activities of daily living will be evaluated using the AMPS. [41] This assessment will be conducted by experienced occupational therapists who participated in a training course for AMPS administration and were calibrated as reliable AMPS raters. Four individual activities of daily living, which the participant is familiar with, will be identified in accordance with the AMPS assessor. Therefore, the participant will select the tasks from the AMPS manual including standardized activities of daily living. The selected tasks must be part of the participant’s everyday life. Two of the chosen four tasks will be performed in the assessment. For the evaluation of individual’s performance, 16 motor and 20 process skill items will each be evaluated by the AMPS rater on a 4-point scale. 4 points are defined as a performance which is conducted effortlessly and safely whereas 1 point is given for performances which for example hold serious deficits concerning safety and efficiency [41]. Higher positive scores are indicative of higher ability. AMPS will be performed at T_0_ and repeated after the training intervention at T_3_.

##### Cardiorespiratory fitness

Cardiorespiratory fitness will be assessed through a progressive CPET performed at baseline on a cycle ergometer (Ergoline 800, Germany) immediately before group allocation because results will be used as stratification factor. CPET will be repeated at T_3_ 48 h after the last exercise session. Details on the protocol can be found elsewhere [42]. The individual cardiorespiratory fitness level is monitored by direct and continuous measurements (breath by breath) of peak oxygen consumption (VO2_peak_) by ergospirometry (PanGas CPX, Germany).

VO2_peak_ is defined as the highest VO2 value when the following criteria are attained: respiratory equivalent ratio > 1.10; peak heart rate within 10 min-1 of age predicted maximum and rating of physical exertion (RPE) > 8.5 as reported by Wassermann et al. [43].

##### Fatigue

Fatigue will be assessed with the multidimensional FSMC scale [44] before CPET under resting conditions. FSMC has defined cut-off scores to classify mildly, moderately and severely fatigued patients. Cut-off for fatigue for the total score is set at 43 and at 22 for the motoric and cognitive sub-scores. The scale has good test-retest reliability and has been translated into German. The fatigue score will be used as a stratification factor, hence, it will be assessed before allocation and repeated at T_3_.

##### Depression and anxiety

Depression and anxiety will be assessed at T_0_ and repeated at T_3_ with the Hospital Anxiety and Depression Scale (HADS) for adults with physical ailments [45]. The questionnaire consists of 14 items, 7 for anxiety and 7 for depression, with higher scores indicating more severe anxiety or depression.

##### Healthcare-related quality of life

Healthcare-related quality of life will be assessed using the Patient-Reported Outcome Measurement Information System (PROMIS) short form Global-10 [46] at T_0_ and will be repeated at T_3_. The PROMIS Global-10 consists of 10 items that separately measure different aspects of perceived symptoms and functioning.

### Safety

Tolerability (safety) of the 3-week training program, and of each bout of exercise for persons with MS, will be monitored by continuous heart-rate measurements. Heart rate (beats per minute) during and after the exercise sessions in both intervention groups should not exceed 220 minus age. This prevents worsening of sensory symptoms and allows normalization within half an hour after the exercise sessions. Moreover, the dropout-rate will be captured in total and for each group.

All severe adverse events - that include i) severe cardiovascular and pulmonary diseases (renal failure, hepatic dysfunction, cardiovascular disease) and ii) severe cardiovascular exacerbations (e.g. RR > 240/120, HR above the age predicted maximum of 220-Age) during training – will be directly reported to the ethical committee.

### Sample size calculation

The required sample size to evaluate the between-group effect of the exercise-interventions (HIIT vs. ST) on the primary endpoint (proportion of Tregs in blood samples of persons with MS) in an analysis of covariance model (ANCOVA) was estimated using G*Power 3.1.9.2 [47]. The following parameters were defined for power analysis: medium effect size of d = .5, alpha of 5% and power (1-β) of 80%. As covariate in the model participants’ proportion of Tregs at baseline will be used. Sample size needed to meet the above stated suppositions can be calculated according to the formula described by Borm and colleagues [48] (1-ρ2)*n. An estimated association between participants’ baseline levels and at T1 of ρ = .7 was used. Calculation of required sample size revealed 33 participants in each group (total *N* = 66). A 10% drop-out rate was expected. This estimation was done according to the attrition rates in previous similar studies [11]. Against this background, we will recruit 72 participants in total.

### Statistics

An intention-to-treat analysis will be conducted. However, data will also be analyzed per protocol. In order to determine potential between-subjects factor effects (HIIT vs ST) at T_3_ on primary and secondary outcomes, separate univariate ANCOVAs will be used. The effects of between-subjects factor acute exercise-intervention (HIIT vs. ST), within-subjects factor measurement time-point (T1 vs. T2) and their interaction on soluble factors will be investigated with 2 × 2 mixed ANCOVAs. Covariation of participants’ levels of soluble factors at T_0_ will be considered. Post hoc Bonferroni corrected pairwise comparisons will be conducted in case of significant main effect of within-subjects factor measurement time-point. To determine if groups differ at T_1_ and/or at T_2_ simple effects analyses will be performed. ANCOVA assumptions will be explored. As measure of effect size Eta-square and Cohen’s d values, respectively, will be calculated. Finally, bivariate correlation analyses will be conducted to determine potential associations between changes in biomarkers (immune status/soluble factors) and cardiorespiratory fitness/cognitive performance/patient-reported outcomes from T_0_ to T_3_.

Statistical analyses will be performed by a statistician blinded to treatment groups coded as 1 or 2. Statistical analyses will be conducted using SPSS 25® (IBM®, Armonk, NY, USA). A result will be considered significant at *p*-value equal to or less than .05.

## Discussion

This study will provide new information on whether the immune-modifying anti-inflammatory effects of exercise, which have previously been described in other populations, occur in persons with MS. We will combine different methods (Flow cytometry, ELISA) and markers (Immune cell subsets, cytokines and Tryptophan metabolites), instead of assessing only a few soluble factors. This will enable us to identify or verify potential mechanism of exercise-induced changes in immune function which are so far only hypothetical, but which are potentially very relevant for people with MS.

Furthermore, the implemented measurement time points, immediately before and after the first exercise session, will extend knowledge of acute exercise-induced effects on immune signalling, according to the intervention (HIIT vs. ST). Examining both acute and chronic effects of exercise within one sample constitutes a robust methodological approach, which allows investigation of any interaction between short- and long-term alterations in the immune functions.

Recent studies have shown that regular physical activity has positive effects on motor and cognitive function in persons with MS [1]. However, the potential mechanisms of exercise induced changes in immune function remain theoretical constructs as no evidence is available that the achieved improvements will impact the everyday life of the person. Here, the additional evaluation of individual activities of daily living and the required motor and process skills (AMPS [41]) will provide new insights about the potential predictors (biomarkers) that strongly impact persons related quality of life. Further the influence of varying exercise intensities will provide more detailed recommendations for rehabilitative training programs.

## Abbreviations

ADL: Activities of daily living
AMPS: Assessment of Motor and Processing Skills
ANCOVA: Analysis of Covariance
BBB: Blood brain barrier
BICAMS: Brief International Cognitive Assessment for Multiple Sclerosis
BVMT-R: Brief Visuospatial Memory Test revised
CPET: Cardiopulmonary exercise test
EDSS: Expanded disability status scale
ELISA: Enzyme-linked immunosorbent assay
FSMC: Fatigue scale of motor and cognitive functions
HADS: Hospital anxiety and depression scale
HIIT: High-intensity interval training
HR_max_: Heart rate maximum
IDO-1: Indoleamine 2,3-dioxygenase-1
IL: Interleukin
KAT: Kynurenine aminotransferase
KYN: Kynurenine
KYNA: Kynurenic acid
min: minutes
MMP: Matrix metalloproteinase
MS: Multiple sclerosis
PROMIS: Patient-Reported Outcome Measurement Information System
QUIN: Quinolinic acid
rpm: revolutions per minute
SDMT: Symbol Digit modalities test
ST: Moderate continuous training (standard therapy)
TAP: Test battery for attentional performance
TDO: Tryptophan 2,3-dioxygenase
TGF-ß: Transforming growth factor-beta
T_regs_: regulatory T-cells
TRP: Tryptophan
VLMT: Verbal learning and memory test
VO2_peak_: Peak oxygen consumption
